# Dexmedetomidine versus propofol on the sedation of pediatric patients during magnetic resonance imaging (MRI) scanning: a meta-analysis of current studies

**DOI:** 10.18632/oncotarget.22271

**Published:** 2017-11-01

**Authors:** Qiang Zhou, Lingli Shen, Xinxian Zhang, Jiong Li, Yong Tang

**Affiliations:** ^1^ Department of Radiology, XuZhou Children's Hospital, Xuzhou, Jiangsu 221006, China; ^2^ Department of Neurology, The Tenth Ward, XuZhou Children's Hospital, Xuzhou, Jiangsu 221006, China

**Keywords:** magnetic resonance imaging, dexmedetomidine, propofol, sedation, meta-analysis

## Abstract

Magnetic resonance imaging (MRI) is a widely applied diagnostic approach for detection of pediatric diseases. Sedatives are commonly used to acquire the accurate MRI images. Dexmedetomidine and propofol serve as sole or combined sedatives in pediatric MRI scanning. This meta-analysis aimed to compare the efficacy of dexmedetomidine and propofol in children ubdergoing MRI. Pubmed, Cochrane Library and Web of Science were searched up to June, 2017. Onset of sedation time, recovery time, sedation time, MRI time, MRI quality and emergence delirium were analyzed. 6 studies with 368 subjects were enrolled in this meta-analysis. The pooling data showed that propofol had a shorter onset of sedation time (WMD: 6.05, 95% CI: 3.12 – 8.98, *P* < 0.0001) and recovery time (WMD: 1.01, 95% CI: 0.36–1.67, *P* < 0.001) than dexmedetomidine. But for sedation time and MRI scanning time, there were no differences between the two groups (sedation time: *P* = 0.29; MRI scanning time: *P* = 0.50). There were no significance between dexmedetomidine and propofol on MRI quality (MRI quality 1: *P* = 1.00; MRI quality 2: *P* = 0.68; MRI quality 3: *P* = 0.45). Two studies using Pediatric Anesthesia Emergence Delirium (PAED) to assess emergence delirium 10 minutes after awakening showed that propofol had a lower PAED than dexmedetomidine (WMD: 2.57, 95% CI: 0.15–5.00, *P* = 0.04). Thus, propofol should be encouraged in pediatric patients undergoing MRI for its better sedative effects and a low incidence of emergence delirium.

## INTRODUCTION

Magnetic resonance imaging (MRI) has become a widely applied diagnostic tool for a series of pediatric diseases [1–3]. This non-invasive, accurate but time-consuming diagnostic approach requires the pediatric examinees to fully cooperate without motion during the operation [4]. Then sedation is usually necessary to accomplish it. Multiple sedative drug regimens have been adopted to achieve satisfactory sedation level. Among them, dexmedetomidine and propofol are commonly used in clinical practice for their specific efficacy and safety characteristics [5, 6]. Both of them have a short sedation and recovery time. Emergence delirium is a common complication in clinical observation [7]. Although several studies have compared the two drugs in pediatric patients undergoing MRI in terms of the above items. A meta-analysis was needed to evaluate the effects of dexmedetomidine and propofol in pediatric MRI imaging.

## RESULTS

### Flow of the included studies

**Figure 1:**
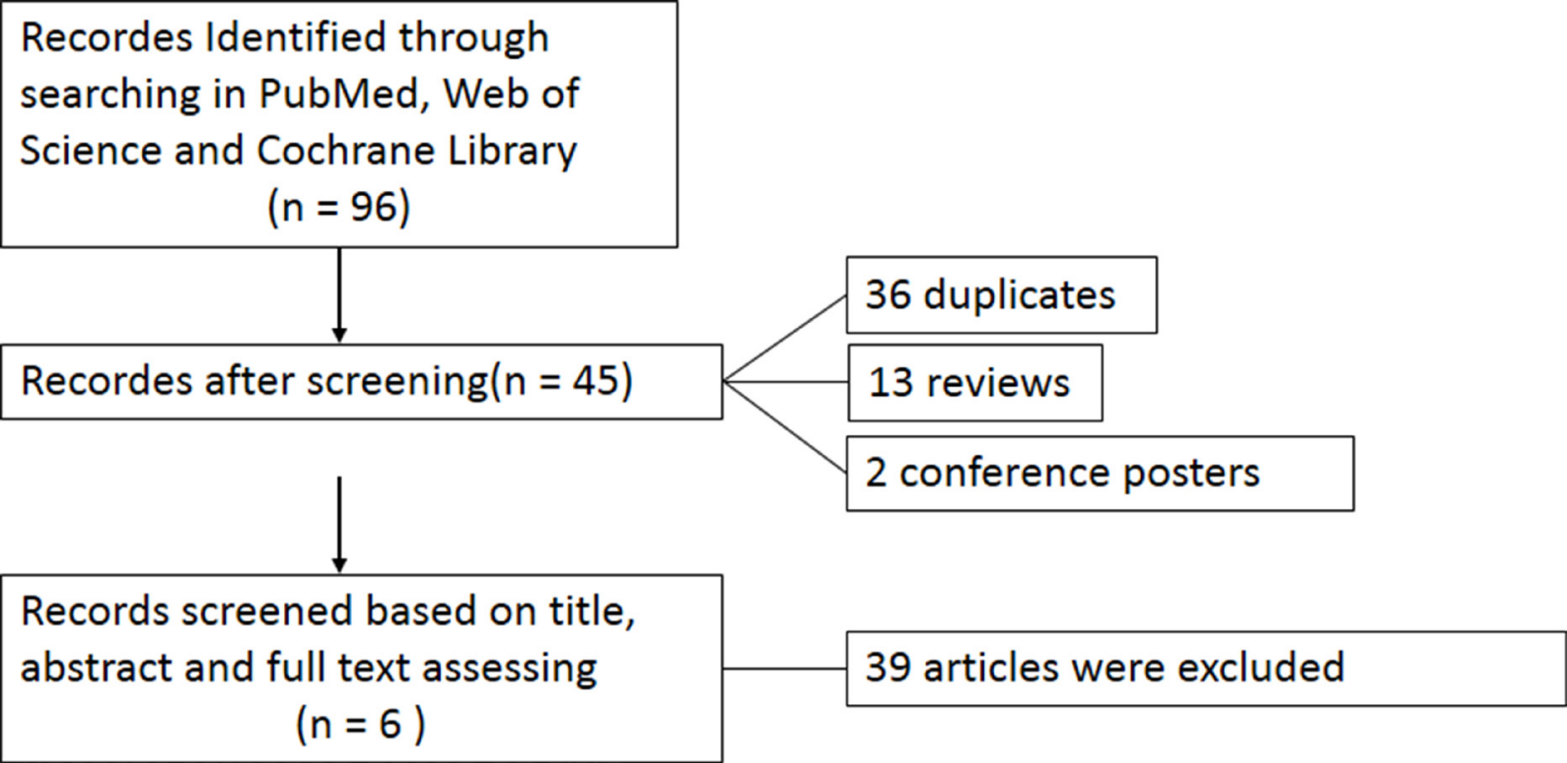
Flow diagram of the meta-analysis

As shown in Figure 1, 96 potential studies were found through searching. Then after careful and thorough screening of the abstracts and whole texts, 36 duplicates, 13 reviews, 2 conference poster were excluded. 38 articles didn't compare the effects between dexmedetomidine and propofol, or provide the data that we want. 1 article was retracted by the journal. They were also excluded. Therefore, 6 studies [8–13] were included with a total of 368 patients fulfilling the inclusion criteria.

### Study characteristics and quality assessment

The articles included were published from 2006 to 2017. 3 were from USA, and the rest 3 came from Turkey, Singapore and India, respectively. There were 186 and 182 patients in dexmedetomidine and propofol, respectively. 1 study used functional MRI, and 5 studies applied MRI scanning. Two studies assessed the sedation level with Ramsay sedation scale, but the other studies didn't indicated it. The baseline characteristics were displayed in Table 1, including age, weight, sex and sedation protocols.

**Table 1:** The baseline characteristics of the included studies

		Dexmedetomidine				
Year	Author	Age	Weight	Sex	Intervention	No.
2006	Koroglu	4 ± 1.88	14 ± 4.14	17	1 ug/kg initial dose followed by continuous infusion of 0.5 ug/kg/h	30
2012	Bernal	6	NA	14	continuous infusion of mean dose 1.8 ug/kg/h	24
2014	Wu	-	-	-	2 ug/kg followed by continuous infusion of 2 ug/kg/h	46
2015	Bong	3	14	24	a single dose of 0.3 ug/kg	40
2016	Watt	4.6 ± 0.8	16.8 ± 5.0	13	1 ug/kg followed by 1 ug/kg/h infusion	16
2017	Kamal	5.2 ± 2.69	16.41 ± 6.21	12	2 ug/kg for 10min followed by continous infusion of 1 ug/kg/h	30
			**Propofol**			
		Age	Weight	Sex	Intervention	No
		3 ± 2.03	14 ± 4.57	10	3 mg/kg initial dose followed by a continuous infusion of 100 ug/kg/min	30
		6.16	NA	9	continuous infusion of mean dose 97.9 ug/kg/h	19
		-	-	-	2 mg/kg and followed by continuous infusion of 200 ug/kg/min	49
		4	15.2	24	a single dose of 1 mg/kg	39
		5.1 ± 1.1	18.2 ± 4.1	10	infusion at 300 ug/kg/min for 10 mins and reduced to 250 ug/kg/min	15
		4.15 ± 2.32	14.86 ± 5.49	14	1 mg/kg bolus followed by continuous infusion of 100 ug/kg/min	30

NA, Not applicable.

### Meta-analysis outcomes

**Figure 2:**
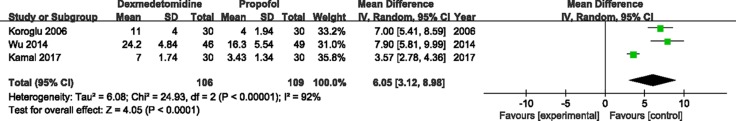
Forest plot and meta-analysis of sedation time

**Figure 3:**
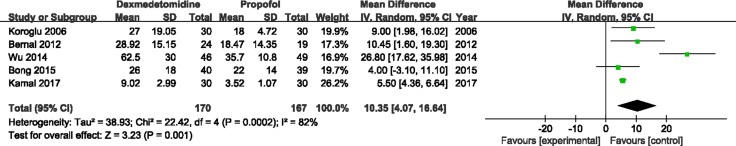
Forest plot and meta-analysis of recovery time

**Figure 4:**
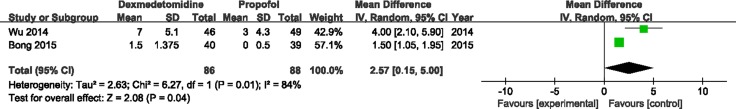
Forest plot and meta-analysis of PAED PAED, Pediatric Anesthesia Emergence Delirium.

The sedation effects, including onset of sedation time, recovery time, discharge time, MRI scanning time were displayed in Supplementary Table 1. The pooling data showed that propofol had a shorter onset of sedation time (WMD: 6.05, 95% CI: 3.12–8.98, *P* < 0.0001) (Figure 2) and recovery time (WMD: 1.01, 95% CI: 0.36–1.67, *P* < 0.001) (Figure 3) than dexmedetomidine (Table 2). But for sedation time and MRI scanning time, there were no differences between the two groups (sedation time: *P* = 0.29; MRI scanning time: *P* = 0.50) (Table 2) (Supplementary Figures 1 and 2). As shown in Supplementary Table 2, 2 studies assessed MRI image quality using a three-point scale (1 = no motion; 2 = minor movement; 3 = major movement necessitating another scan). The pooling analysis showed that there were no significance between dexmedetomidine and propofol (MRI quality 1: *P* = 1.00; MRI quality 2: *P* = 0.68; MRI quality 3: *P* = 0.45) (Table 2) (Supplementary Figures 3, 4 and 5). Two studies assessing Pediatric Anesthesia Emergence Delirium (PAED) 10 minutes after awakening showed that propofol had a lower PAED than dexmedetomidine (WMD: 2.57, 95% CI: 0.15–5.00, *P* = 0.04) (Table 2) (Figure 4) (Supplementary Table 3).

**Table 2:** Meta-analysis of the included studies

	Study No.	WMD (95% CI)	*P*	Study Heterogeneity			
				χ^2^	df	I^2^, %	*P*
Onset of sedation time	3	6.05 (3.12, 8.98)	< 0.0001	24.93	2	92	< 0.00001
Recovery time	5	10.35 (4.07, 16.64)	0.001	22.42	4	82	0.0002
Sedation time	4	2.15 (−1.82, 6.12)	0.29	4.00	3	25	0.26
MRI time	3	−1.37 (−5.38, 2.64)	0.50	6.95	3	57	0.07
**MRI quality**							
1	2	1.00 (0.47, 2.12)	1.00	0.15	1	0	0.70
2	2	−0.03 (−0.19, 0.13)	0.68	0.00	1	0	1.00
3	2	1.80 (0.39, 8.32)	0.45	NA	NA	NA	NA
PAED	2	2.57 (0.15, 5.00)	0.04	6.27	1	84	0.01

MRI, Magnetic resonance imaging; PAED, Pediatric Anesthesia Emergence Delirium; WMD, weighted mean difference.

### Heterogeneity and bias

Sensitivity analysis was made in comparison with significant heterogeneity among studies (Table 2). There were less than 10 high-quality studies in our meta-analysis, a publication bias assessment cannot be performed accurately.

## DISCUSSION

This meta-analysis compared the effects of dexmedetomidine and propofol among pediatric patients undergoing MRI using the available medical data. The study discovered some findings: there is enough evidence in support of the clinical application of propofol for a shorter onset of sedation time and recovery time than dexmedetomidine. No significance were found between the two interventions of sedation time, MRI scanning time and MRI image quality. Propofol seemed to have a lower rate of emergence delirium.

We were fully aware of a previously published meta-analysis [14] in 2015 on the same subject. However, we found that one article included in that study was retracted, making their findings not reliable. This then encouraged the establishment of this meta-analysis. The present study is of great importance to demonstrate the comparison of dexmedetomidine and propofol sedation in children undergoing MRI. Although the conclusions of the two are quite similar, it is vital to take the current analysis to exclude the potential discrepancy caused by the retracted study. This would help the purity and accuracy of understanding of dexmedetomidine and propofol.

MRI scanning is a useful diagnostic imaging tool in pediatric patients for its high accuracy and non-radiation [15]. However, it requires the examinees’ full cooperation to be motionless. It is difficult for pediatric sufferers to follow the instructions during scanning, making the examination hard to finish [16]. Therefore, anesthesiologists and other clinical operators are encouraged to use specific sedatives in practice. Dexmedetomidine and propofol are both commonly used sedatives in children undergoing MRI examination for their efficacy and safety [5, 17]. Dexmedetomidine is a potent α_2_-adrenoceptor agonist. It has sedative, analgesic and opioid-sparing effects and is suitable for short- and long-term sedation. It's used in pediatric patients due to its efficacy and lack of adverse respiratory events [18, 19]. Propofol is an intravenous agent used for induction and maintenance of anesthesia in children. It has many pharmacological advantages over other agents such as rapid effect, short action and few side effects through the interaction with various neurotransmitter receptors [6, 20]. Several studies had evaluated and compared them in pediatric subjects during MRI in recent years. Koroglu et al. [6, 20] believed that dexmedetomidine and propofol produced enough sedation in children undergoing MRI. And dexmedetomidine would be a reliable alternative to propofol for its less side effect incidences. Also no sifgnificance were found between the two of adverse events by Bernal et al. [9]. However, that study was done using functional MRI, which might be a factor for the results. And it didn't compare the two on sedative effects. Some researchers discovered a quite different fact in clinical application. Propofol yielded overall better ourcomes than dexmedetomidine in terms of timeliness [10].

Emergence delirium is a common and well-recognized complication occurring in children with general anesthesia [7]. In addition, the incidence of this complication is relatively high following pediatric sedation for MRI. PAED scale is developed to quantify it by assessing restlessness, eye contact, inconsolability, purpose actions and consciousness [21]. Both dexmedetomidine and propofol are capable of reducing emergence delirium in the practice [22–24]. Bong et al. compared the two sedatives and found that there were no differences of the two on the incidence of emergence delirium [22–24]. In this study, two studies investigated PAED scores 10 minutes after awakening. Consistent with the previous studies, no differences were found after pooling analysis.

There were some limitations in this study. First, the limited number of studies included in this meta-analysis would hinder the application of our conclusion. Second, the sedation procedure of each drug in all the included studies were not the same. This might cause heterogeneity. Third, we could not thoroughly examine physiological parameter changes during scanning for the lack of the relevant data. Further analysis on this topic should be done in the future. This will facilitate the clinical selection of the suitable sedative drug to avoid potential unexpected effects.

We believe that propofol should be encouraged in pediatric patients undergoing MRI for its better sedative effects and a low incidence of emergence delirium.

## MATERIALS AND METHODS

### Literature search strategy

Preferred Reporting Items for Systematic Reviews and Meta-analysis (PRISMA) [25] and Meta-analysis of Observational Studies in Epidemiology [26] recommendations were used for study reporting. This study was based on previously studies; thus, ethical approval and patient consent were not required. A computerized search of the Pubmed, Web of Science, and Cochrane Library databases was performed through to June 2017, without restriction on the language or publication type, using keywords as following: pediatric, child, children, adolescence, adolescent, nuclear resonance imaging, magnetic resonance imaging, MRI, dexmedetomidine, Precedex, Dexmedetomidine Hydrochloride, Propofol, Diprivan and Disoprofol.

### Inclusion criteria and exclusion criteria

In order to insure the homogeneity of the studies, the following criteria should be met: (1) all the patients were ≤ 18 years old, and scheduled to accomplish MRI with general anesthesia; (2) the study used dexmedetomidine and propofol as the sole sedative agent and compared the two in the sedation process; (3) the information, including onset of sedation time, recovery time, sedation time, MRI scanning time, MRI quality assessment and adverse effects were provided. Also the exclusion criteria was given as following: (1) patients were accompanied with developmental delay, cognitive decline, severe central nervous system disorders that might influence the anesthesia effects; (2) studies used dexmedetomidine or propofol plus other sedative reagents for anesthesia. Any disagreement over the selection process was resolved after consensus-based discussion.

### Data extraction and quality assessment

Data from the included studies were extracted and summarized independently by Qiang Zhou and Lingli Shen. Any disagreement was resolved by consensus-based discussion and determined by Xinxian Zhang. The outcomes were the comparison of sedation effects (onset of sedation time, recovery time and sedation time), MRI scanning quality (MRI time and MRI image quality) and the incidence of emergence delirium PAED score 10 minutes after awakening) between dexmedetomidine and propofol. The quality of cohort studies was assessed by the Cohort Studies Version of Newcastle-Ottawa Quality Assessment Scale, which consists of three factors: Patient selection, comparability of the study groups, and assessment of outcome. A score of 0–9 was allocated to each study. Studies that achieved six or more stars were considered to be of high quality.

### Statistical analysis

Meta-analysis was performed on studies that provided data on outcomes of patients using the software package Stata 14. The weighted mean difference (WMD) was used to compare continuous variables. All results were reported with 95% confidence intervals (CIs). Statistical heterogeneity was assessed using the I^2^ statistic, which describes the proportion of total variation that is attributable to differences among trials rather than sampling error (chance). An I^2^ value of < 25% was defined to represent low heterogeneity, a value between 25% and 50% was defined as moderate heterogeneity and > 50% was defined as high heterogeneity. The random-effects model was used if there was high heterogeneity between studies. Otherwise, the fixed-effects model was used. Publication bias was calculated using Egger's test.

## SUPPLEMENTARY MATERIALS FIGURES AND TABLES


