# Supplementary material for: Dexmedetomidine versus propofol on the sedation of pediatric patients during magnetic resonance imaging (MRI) scanning: a meta-analysis of current studies
Source: Oncotarget. 2017 Nov 1;8(60):102468–73. doi: 10.18632/oncotarget.22271 (PMC5731972; doi:10.18632/oncotarget.22271)
Supplement: Supplementary file 1 [file oncotarget-08-102468-s001.pdf]

Dexmedetomidine versus propofol on the sedation of pediatric patients during magnetic resonance imaging (MRI) scanning: a meta-analysis of current studies

SUPPLEMENTARY MATERIALS

Supplementary Table 1: The sedation effects and MRI scanning time

	Dexmedetomidine				Propofol			
Study	onset of sedation time	Recovery time	Sedation time	MRI time	onset of sedation time	Recovery time	Sedation time	MRI time
2006	11 ± 4	27 ± 19.05	47 ± 14.93	22 ± 7.14	4 ± 1.94	18 ± 4.72	46 ± 17.59	25 ± 10.14
2012	-	28.92 ± 15.15	148.83 ± 28.87	-	-	18.47 ± 14.35	126.58 ± 37.04	-
2014	24.2 ± 4.84	62.5 ± 30.0	-	91.5 ± 25.6	16.3 ± 5.54	35.7 ± 10.8	-	81.7 ± 21.7
2015	-	26 ± 18	71 ± 5	-	-	22 ± 14	70 ± 28	-
2016	-	-	-	52 ± 11	-	-	-	58 ± 12
2017	7 ± 1.74	9.02 ± 2.99	30.2 ± 5.26	23.33 ± 4.64	3.43 ± 1.34	3.52 ± 1.07	28.6 ± 4.61	25.18 ± 5.01

MRI, Magnetic resonance imaging

Supplementary Table 2: MRI image quality assessment

	Dexmedetomidine			Propofol		
Study	1	2	3	1	2	3
2006	19	6	5	20	7	3
2012	-	-	-	-	-	-
2014	-	-	-	-	-	-
2015	-	-	-	-	-	-
2016	-	-	-	-	-	-
2017	20	10	0	19	11	0

Supplementary Table 3: PAED 5 min and 10 min after awakening

	Dexmedetomidine		Propofol	
Study	5 min	10 min	5 min	10 min
2006	-	-	-	-
2012	-	-	-	-
2014	8.3 ± 4.5	7.0 ± 5.1	5.6 ± 4.5	3.0 ± 4.3
2015	-	1.5 (0-5.5)	-	0 (0-2)
2016	-	-	-	-
2017	-	-	-	-

PAED, Pediatric Anesthesia Emergence Delirium

Study or Subgroup	Dexmedetomidine Mean	SD	Total	Propofol Mean	SD	Total	Weight	Mean Difference IV, Random, 95% CI	Year
Koroglu 2006	47	14.93	30	46	17.59	30	18.1%	1.00 [-7.26, 9.26]	2006
Bernal 2012	148.83	28.87	24	126.58	37.04	19	3.7%	22.25 [1.98, 42.52]	2012
Bong 2015	71	5	40	70	28	39	16.0%	1.00 [-7.92, 9.92]	2015
Kamal 2017	30.2	5.26	30	28.6	4.61	30	62.3%	1.60 [-0.90, 4.10]	2017
**Total (95% CI)**			**124**			**118**	**100.0%**	**2.15 [-1.82, 6.12]**	

Heterogeneity: Tau² = 4.95; Chi² = 4.00, df = 3 (P = 0.26); I² = 25%

Test for overall effect: Z = 1.06 (P = 0.29)

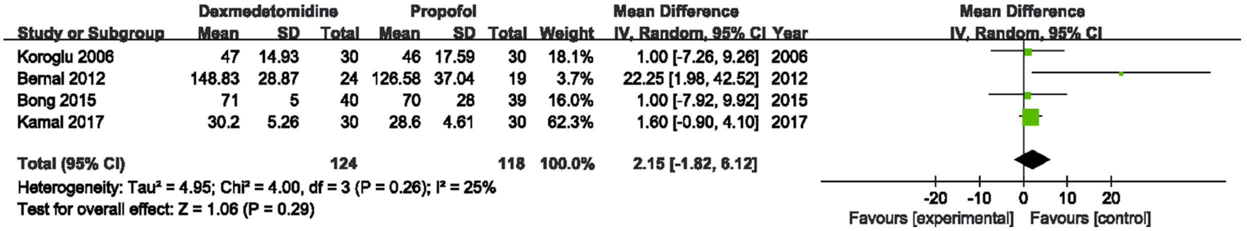

Supplementary Figure 1: Forest plot and meta-analysis of sedation time.

Study or Subgroup	Dexmedetomidine Mean	SD	Total	Propofol Mean	SD	Total	Weight	Mean Difference IV, Random, 95% CI	Year
Koroglu 2006	22	7.14	30	25	10.14	30	30.2%	-3.00 [-7.44, 1.44]	2006
Wu 2014	91.5	25.6	46	81.7	21.7	49	12.9%	9.80 [0.23, 19.37]	2014
Watt 2016	52	11	16	58	12	15	16.2%	-6.00 [-14.12, 2.12]	2016
Kamal 2017	23.33	4.64	30	25.18	5.01	30	40.7%	-1.85 [-4.29, 0.59]	2017
**Total (95% CI)**			**122**			**124**	**100.0%**	**-1.37 [-5.38, 2.64]**	

Heterogeneity: Tau² = 8.75; Chi² = 6.95, df = 3 (P = 0.07); I² = 57%

Test for overall effect: Z = 0.67 (P = 0.50)

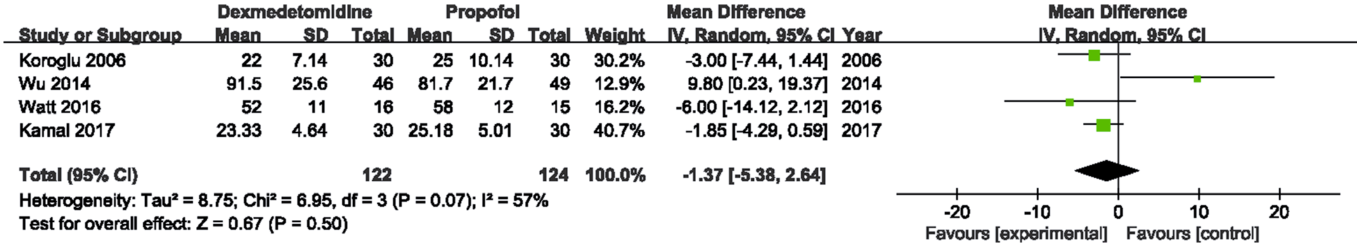

Supplementary Figure 2: Forest plot and meta-analysis of MRI scanning time.

Study or Subgroup	Dexmedetomidine Events	Total	Propofol Events	Total	Weight	Odds Ratio M-H, Fixed, 95% CI	Year
Koroglu 2006	19	30	20	30	53.7%	0.86 [0.30, 2.50]	2006
Kamal 2017	20	30	19	30	46.3%	1.16 [0.40, 3.35]	2017
**Total (95% CI)**		**60**		**60**	**100.0%**	**1.00 [0.47, 2.12]**	
Total events	39		39				

Heterogeneity: Chi² = 0.15, df = 1 (P = 0.70); I² = 0%

Test for overall effect: Z = 0.00 (P = 1.00)

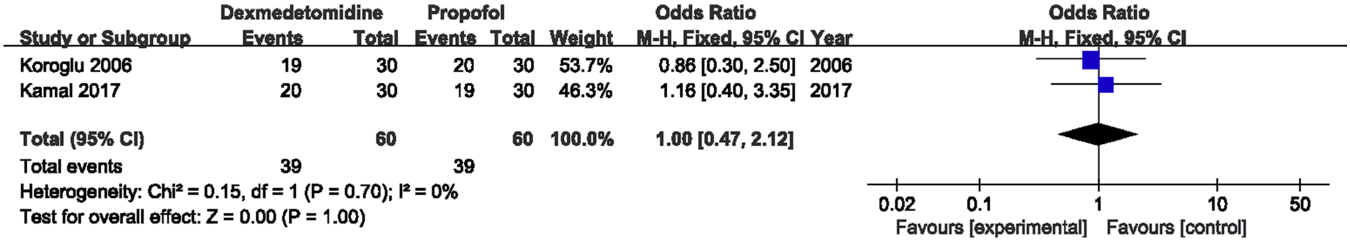

Supplementary Figure 3: Forest plot and meta-analysis of MRI quality 1.

Study or Subgroup	Dexmedetomidine Events	Total	Propofol Events	Total	Weight	Risk Difference M-H, Fixed, 95% CI	Year
Koroglu 2006	10	30	11	30	50.0%	-0.03 [-0.27, 0.21]	2006
Kamal 2017	6	30	7	30	50.0%	-0.03 [-0.24, 0.17]	2017
**Total (95% CI)**		**60**		**60**	**100.0%**	**-0.03 [-0.19, 0.13]**	
Total events	16		18				

Heterogeneity: Chi² = 0.00, df = 1 (P = 1.00); I² = 0%
Test for overall effect: Z = 0.41 (P = 0.68)

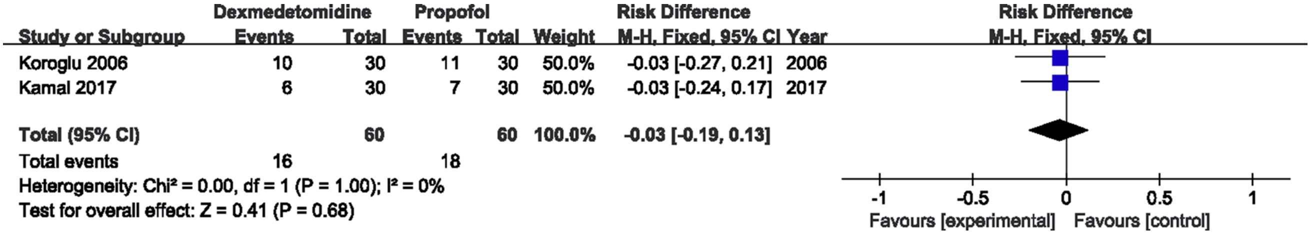

Supplementary Figure 4: Forest plot and meta-analysis of MRI quality 2.

Study or Subgroup	Dexmedetomidine Events	Total	Propofol Events	Total	Weight	Odds Ratio M-H, Fixed, 95% CI	Year
Koroglu 2006	5	30	3	30	100.0%	1.80 [0.39, 8.32]	2006
Kamal 2017	0	30	0	30		Not estimable	2017
**Total (95% CI)**		**60**		**60**	**100.0%**	**1.80 [0.39, 8.32]**	
Total events	5		3				

Heterogeneity: Not applicable
Test for overall effect: Z = 0.75 (P = 0.45)

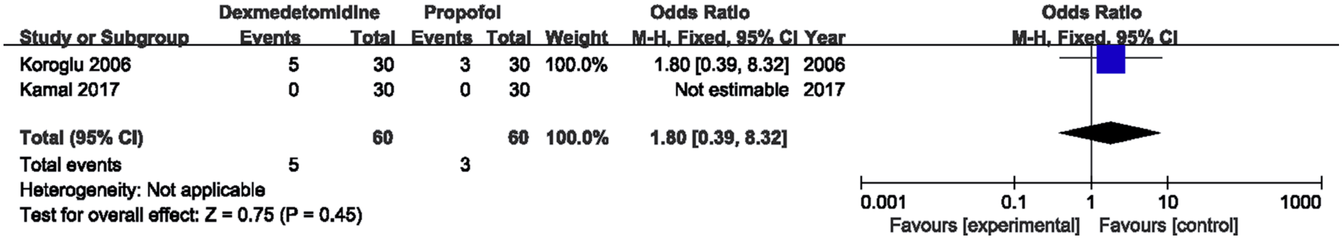

Supplementary Figure 5: Forest plot and meta-analysis of MRI quality 3.
